# Dynamic Bayesian networks to predict loss of kidney function: a cross-institution use case in a large cohort with or at-risk of CKD

**DOI:** 10.1186/s12911-026-03570-6

**Published:** 2026-05-20

**Authors:** Panayiotis Petousis, David Gordon, O. Kenrik. Duru, Keith C. Norris, Katherine R. Tuttle, Susanne B. Nicholas, Alex A. T. Bui

**Affiliations:** 1 Clinical and Translational Science Institute, University of California, 924 Westwood Blvd, Suite 420, Los Angeles, CA 90024 USA; 2 Medical & Imaging Informatics Group University of California, Los Angeles, CA USA; 3 Providence Medical Research Center, Providence Inland Northwest Health, Spokane, WA USA; 4 Division of General Internal Medicine & Health Services Research, Department of Medicine, University of California, Los Angeles, CA USA; 5 Department of Medicine, Division of Nephrology, University of California, Los Angeles, CA USA; 6 Department of Medicine, University of Washington, Seattle, WA USA; 7 Kidney Research Institute and Institute of Translational Health Sciences, University of Washington, Seattle, WA USA

**Keywords:** Machine learning, Chronic kidney disease, ≥40% eGFR decline, Dynamic Bayesian network

## Abstract

**Background:**

Substantial loss of kidney function, measured as ≥40% decline in estimated glomerular filtration rate (eGFR) within a 2-year period, is associated with a tenfold increase in the risk for kidney failure.

**Methods:**

We developed and externally validated dynamic Bayesian networks (DBNs) to predict ≥ 40% eGFR decline using electronic health record (EHR) data from Providence and UCLA Health.

**Results:**

Of 2.25 million patients, with and at-risk for chronic kidney disease (CKD), 6.49% (146,043 individuals) experienced at least one occurrence of ≥40% decline from baseline eGFR over six years of follow-up. The DBNs demonstrated strong predictive performance, as measured by the area under the receiver operating characteristic curve (AUCROC) and average precision (AP), with the highest values observed in the final year (Year 6), ranging from 0.83–0.89 and 0.28–0.37, respectively. A comparison of existing gold-standard CKD-Prognosis Consortium risk equations in real-world clinical settings with missing data demonstrated that DBNs’ performance remained unaffected, while risk equations performed close to random due to their inability to handle missing data. The temporal structure of the DBNs captured longitudinal features changes and their interactions, with the most important observations over time including the urine albumin-creatinine ratio (UACR), the urine protein-creatinine ratio, and hemoglobin A1c. Notably, comparing DBN performance across institutions revealed that training on larger datasets generalized better.

**Conclusion:**

This study offers valuable insights into the development of DBNs using real-world population data from EHRs. The DBNs successfully identified patients at-risk for ≥40% eGFR decline and, despite missing data, offer opportunities for timely intervention and risk mitigation to preserve kidney function.

**Supplementary Information:**

The online version contains supplementary material available at 10.1186/s12911-026-03570-6.

## Background

The incidence of kidney failure has increased by an alarming 41.8% between 2000 and 2019 in the United States (US), with 7% of all Medicare claims now related to this condition [1]. Substantial loss of kidney function [2, 3], represented by a decline in estimated glomerular filtration rate (eGFR) of at least 40% or more, within two consecutive years, is associated with a tenfold higher risk for kidney failure [4, 5]. As such, a ≥ 40% decline in eGFR is used by the US Food and Drug Administration as a surrogate outcome in clinical trials of interventions for chronic kidney disease (CKD). The timely prediction of substantial loss of kidney function would permit early identification of people at-risk of CKD onset and progression, who may benefit from interventions to preserve kidney function, or to proactively plan for kidney replacement therapy, such as dialysis or kidney transplant.

Statistical and machine learning (ML) approaches using electronic health records (EHR) are increasingly used for risk prediction of CKD onset and progression (e.g., the Kidney Failure Risk Equation, KFRE) [6], for the prediction of acute kidney injury (AKI) [7], or to support clinical decision making [8]. However, these existing risk calculators are typically validated on specific patient populations or are predicated on static datasets, with minimal (if any) missing values, rather than on dynamic, longitudinal changes in real-world settings. Thus, these existing calculators have limited utility in practice.

The aim of this study was to develop data-driven CKD onset and progression models using an EHR dataset of >2.2 M patients, at-risk for CKD or with CKD stages 1–5, derived over a 15-year period from two independent US healthcare systems. Specifically, we developed dynamic Bayesian network models (DBNs) that predict ≥ 40% eGFR decline over a continuous 6-year horizon. Unlike many other ML techniques, DBNs excel in explainability and insight generation [9]. Additionally, DBNs combine present and past observations to predict future outcomes over time, while simultaneously inferring missing data. The developed DBNs were externally validated across two large healthcare systems, demonstrating improving performance over the 6-year period and providing insights into factors that affected eGFR change.

Several publications have described the use of random forest (RF) models, combining demographic, laboratory, and other clinical data to predict loss of eGFR. Inaguma et al. [10], used a cohort of 29,466 CKD patients to learn an RF to predict ≥ 30% eGFR decline within two years, showing model AUCROC ranging from 0.69–0.79 in patients with different eGFR trajectories. This model identified hemoglobin, albumin, and C-reactive protein as important features. Using the Mount Sinai BioMe Biobank, Chauhan et al. [11] developed RFs using soluble tumor necrosis factor receptors and the EHR to predict a composite outcome, including a ≥40% sustained eGFR decline. The reported AUCROC for the models ranged from 0.77–0.80. The Klinrisk model [12], a machine learning tool leverages routinely collected laboratory data to predict a sustained ≥ 40% decline in eGFR or kidney failure. The Klinrisk model was developed using a Manitoba, Canada, population and externally validated on a population from Alberta, Canada, with model performance at 2- and 5-year prediction of 0.88 and 0.84 on the internal test set, respectively. On an external cohort, model performance was 0.87 and 0.84, respectively. Further validation in high-risk clinical trial populations, such as the FIDELITY pooled analysis and the CANVAS/CREDENCE trials, further solidified the model’s utility, achieving AUCs up to 0.88 at three years in patients with type 2 diabetes [13, 14]. Subsequent large-scale validation across 4.8 million US adults, including commercial, Medicare, and Medicaid populations, confirmed its robust performance, yielding AUCs of 0.80–0.87 and outperforming the current standard-of-care described for Kidney Disease: Improving Global Outcomes (KDIGO) heatmap categories [15].

Lastly, [16] developed a model that combined clinical and ultrasound imaging features to predict 5-year eGFR decline in patients with kidney transplants. A comparison of AUCROC for the joint imaging-clinical feature model, clinical features alone, and KFRE resulted in 0.81, 0.62, and 0.67, respectively. Grams et al., as part of the CKD-Prognosis Consortium (CKD-PC) developed a logistic regression model [17] predicting ≥ 40% eGFR decline at Year 3 from baseline. The CKD-PC model was created using 43 different cohorts (>1.6 M participants) drawn from research studies, EHRs, and clinical trials worldwide. It uses five variables (age, eGFR, UACR, sex, and diabetes status; diabetes is used only as a flag variable to distinguish between the non-diabetic and diabetic model equations) to predict a 3-year probability of an adverse kidney outcome of ≥40% eGFR decline or kidney failure.

In comparison, we developed DBNs to predict ≥ 40% eGFR decline and externally validated them at two healthcare institutions, UCLA Health and Providence Health. This work provides important contributions to: 1) the prediction of ≥40% eGFR decline using real-world EHR data with missing values; 2) the generation of insight between key clinical variables and kidney progression over time; and 3) the development of a methodology robust to missing data, which was compared against traditional ML models and the CKD-PC model in scenarios with both missing and complete data.

## Methods

In this section, we outline the cohorts, preprocessing methods, and model development methods to build and apply DBNs to predict loss of kidney function. The methodology of this study involved three phases: preprocessing, model development, and evaluation. We used the Center for Kidney Disease Education and Hope (CURE-CKD) Registry, comprising > 2.25 million patients from University of California, Los Angeles Health (UCLA) and Providence Health (PHS) systems, and filtered for individuals with at least 1 year of follow-up, stratifying them by ≥ 40 eGFR decline. Input variables were discretized to facilitate the development of discrete DBNs capable of handling longitudinal data with missing values. We constructed and compared different graphical structures for the DBN of each site, using the expectation-maximization algorithm for training. Finally, the models were assessed through internal and external validation using metrics such as AUCROC and average precision, and their performance was benchmarked against traditional ML models and the established CKD-PC risk model in both complete and missing data scenarios.

## CURE-CKD: an observational EHR dataset for understanding CKD onset and progression

**Fig. 1:**
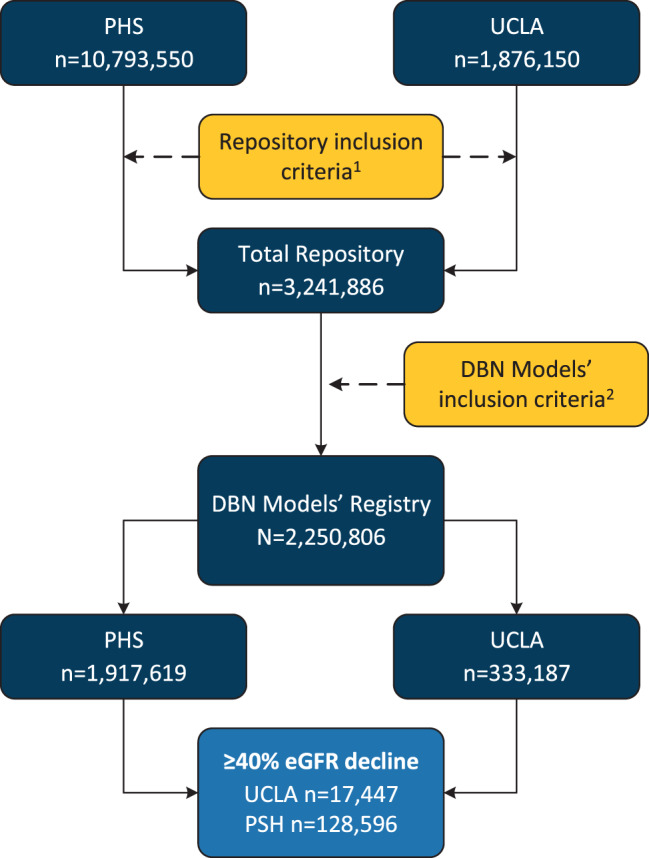
STROBE diagram; ‘1’ (repository inclusion criteria) and ‘2’ (DBN models’ inclusion criteria): more details on criteria in Supplemental Materials 1 section 1.1. Abbreviations: UCLA, University of California, Los Angeles Health; PHS, Providence Health System; DBN, dynamic Bayesian Network; eGFR (estimated glomerular filtration rate)

We utilized data from the CURE-CKD Registry [18], which contains EHR information on patients from the UCLA and PHS systems. Individuals are included in the registry based on a diagnosis of CKD (per International Classification of Disease 9/10 diagnosis code), or eGFR < 60 mL/min/1.73 m^2^; or at-risk for developing CKD based on pre-diabetes, diabetes mellitus, or hypertension by ICD-9/10 code, vital signs, medications, or lab values [19]. Registry information over 15 years (1/1/2006–12/31/2020) resulted in 2,250,806 unique patients (UCLA, *n* = 333,187; PHS, *n* = 1,917,619). The prevalence of at least one eGFR measurement with a ≥ 40% decline in the follow-up period was *n* = 17,447 (5.24%) and *n* = 128,596 (6.71%) for UCLA and PHS, respectively; and 6.49% for UCLA+PHS collectively (Fig. 1). Outliers were assigned missing values based on clinical criteria, and only patients with at least one year of follow-up were included in our modeling and evaluation tasks. Specific details on the inclusion criteria are in Supplemental Materials 1, Section 1.1. After filtering, PHS contributed 85%, and UCLA contributed 15% to the cohort.

In defining kidney function trajectories, we accounted for the physiological volatility of eGFR. Rather than treating all ≥ 40% declines as irreversible progression, our methodology incorporates the possibility of subsequent eGFR recovery. This allows modeling both chronic progression and resolved acute episodes (AKI/AKD) within the CURE-CKD cohort.

**Fig. 2:**
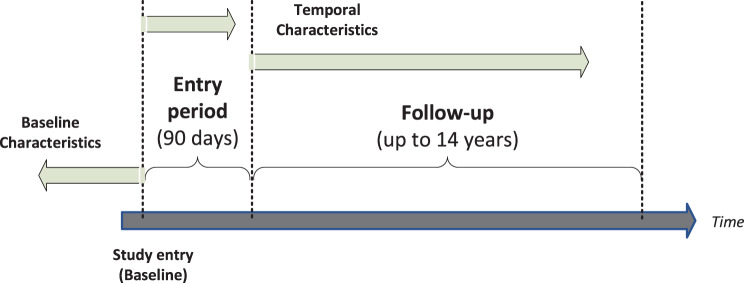
CURE-CKD data generation timeline. Study entry (baseline) represents the day of entering the study. Baseline characteristics represent the variables listed in Table 1. The first 90 days after study entry are the entry period. The follow-up period is a 14-year period. Temporal characteristics represent the variables observed in the entry period and follow-up period. These variables are listed in Table 2

Every patient captured in the CURE-CKD registry is represented by three distinct periods (Fig. 2): 1) *study entry (baseline),* at which multiple initial observations are recorded; 2) *entry period*, the immediate 90-day period following baseline entry during which clinical observations are collected; and 3) a *follow-up period* of up to 14 years, in which all clinical variables and observations are monitored annually. The study was approved by PHS (Washington, Montana, Oregon, New Mexico, Alaska, and California; IRB #SPK2043) and UCLA Health (IRB #20-000056) institutional review boards.

## Dataset preprocessing

**Fig. 3:**
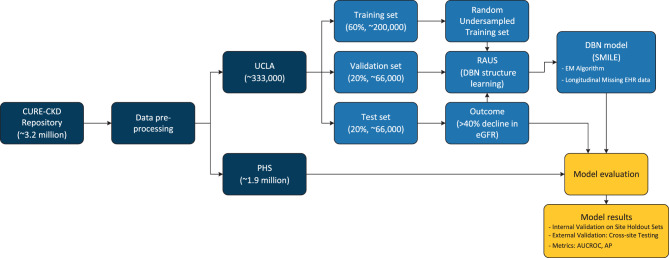
Model evaluation process. In this example, UCLA is the basis for creating the training, validation, and internal testing set in this diagram. The PHS cohort is then used as an external validation set. This framing is reversed when the PHS cohort is used to learn the DBN. When the cohorts are combined, there is no external validation. Abbreviations: RAUS (ranking approaches for unknown structures), DBN (dynamic Bayesian network), SMILE (Structural modelling, Inference, and learning Engine)

The dataset was partitioned according to the health system (i.e., UCLA, PHS, UCLA+PHS) (Fig. 3). Each split was further stratified based on the outcome of ≥40% eGFR decline at least once during follow-up, and subsequently randomly split into training (60%), validation (20%), and holdout testing sets (20%). Stratification was based on maintaining the same proportion of ≥40% eGFR decline cases across each group. Each training set was randomly under-sampled to a 1:1 ratio for balanced learning of ≥40% eGFR decline cases to non-decliners (i.e., individuals that do not experience a ≥ 40% eGFR decline), as previously described [20, 21]. To build the DBNs, the input variables were discretized into ranges or categorical variables. For continuous variables (e.g., eGFR), we employed minimum description length discretization [22]. See Supplemental Materials, 1 Sect. 1.2 for additional details.

## Building the dynamic Bayesian networks

**Fig. 4:**
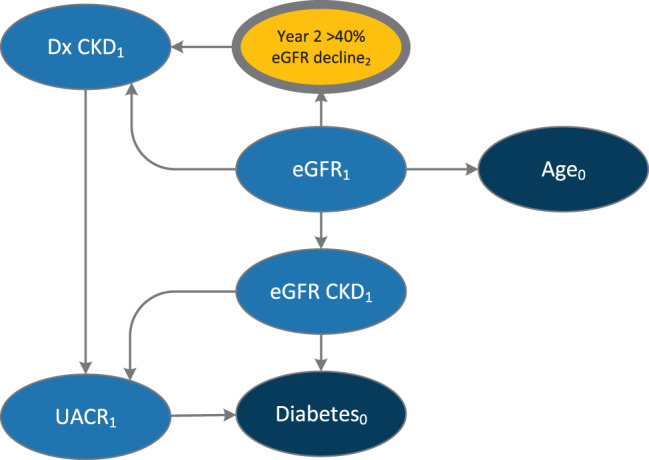
Example dynamic belief network where the probability of eGFR decline is based on several observations over time. Nodes in this graph represent model variables, while arrows between the nodes denote conditional dependence. Abbreviations: dx CKD (chronic kidney disease identified from diagnosis code), eGFR CKD (chronic kidney disease identified from estimated glomerular filtration rate), DM (diabetes mellitus), UACR (urine albumin-creatinine ratio), UPCR (urine protein-creatinine ratio). _0_: entry/baseline data, _1_: study entry period data, _2_: Year 1 data

Briefly, a Bayesian network (BN) is a type of ML model that captures a joint probability distribution across multiple variables through a set of conditional relationships. [23] Fig. 4 demonstrates an example of the computation of the probability of ≥40% eGFR decline (*t* = 2) on a patient at the first year of follow-up based on a simplified DBN, where the computation of the conditional probability is based on the available evidence for a given patient at baseline (*t* = 0; age, diabetes status) and the prior year (*t* = 1; eGFR, CKD status, urine albumin-to-creatinine ratio (UACR) value):

$$\eqalign{& P( \ge 40\% {\rm{eGFRdeclin}}{{\rm{e}}^{\left( {t = 2} \right)}}{\rm{|}}eGF{R^{t = 1}}, \cr & eGFRCK{D^{t = 1}},D{M^{t = 0}},UAC{R^{t = 1}}, \cr & DxCK{D^{t = 0}},Ag{e^{t = 0}}) \cr} $$

We viewed the problem of predicting rapid CKD progression as one of determining the probability of a future ≥ 40% eGFR decline based on *current* and *past* observations of an individual. We used DBNs to model the annual loss of kidney function for the 6-year follow-up period. To constrain the scope of features employed in the model, clinically relevant variables for CKD progression were prespecified [19]. Tables 1 and 2 list all variables used in the models. For a DBN, two subtasks were involved in model learning: first, determining the graph structure (connections between variables), characterizing the dependencies at a given point in time and over time; and second, calculating the associated conditional probabilities. We aimed to determine and compare three graph structures based on site-specific associations: 1) a UCLA-specific model (i.e., trained using only UCLA data); 2) a PHS-specific model (i.e., trained using only PHS data); and 3) a joint UCLA+PHS model (i.e., using data from both health systems). Each site model consists of three interconnected substructures, each learned from a specific time (baseline, entry period, and follow up period) period of the study, as shown in Fig. 2. Supplemental Materials, Sect. 1.3, outlines the generation of these graph structures. Following the graphs’ derivation, the models were trained using the expectation-maximization algorithm [24].

**Table 1:** Categorical variables, proportions, and percentage of missing data

Variables	Categories	Proportions (%)	Missing values
Site	UCLA	14.80	0.00
	PHS	85.20	
Sex	Male	45.44	0.00
	Female	54.56	
Race and Ethnicity	White	68.47	0.00
	Hispanic	3.63	
	Black	4.66	
	Asian	6.33	
	Native American	0.95	
	Hawaiian	0.60	
	Other	9.72	
	Not Categorized	5.64	
RUCA 4 code class	Isolated	2.30	0.97
	Large Rural	5.12	
	Small Rural	2.41	
	Urban	89.20	
Study Entry CKD from eGFR lab	No	83.17	0.00
	Yes	16.83	
Study Entry CKD from diagnosis code	No	97.06	0.00
	Yes	2.94	
Study Entry CKD from Albuminuria/Proteinuria	No	98.57	0.00
	Yes	1.43	
Study Entry DM	No	85.13	0.00
	Yes	14.87	
Study Entry Pre-DM	No	91.29	0.00
	Yes	8.71	
Study Entry Hypertension	No	65.53	0.00
	Yes	34.47	
Study Entry ACEIARB medication	No	87.09	0.00
	Yes	12.91	
Study Entry NSAID medication	No	87.66	0.00
	Yes	12.34	
Study Entry PPI medication	No	91.32	0.00
	Yes	8.68	
Year 1 ≥ 40% eGFR decline	No	99.20	0.00
	Yes	0.80	
Year 2 ≥ 40% eGFR decline	No	98.96	0.00
	Yes	1.04	
Year 3 ≥ 40% eGFR decline	No	98.84	0.00
	Yes	1.16	
Year 4 ≥ 40% eGFR decline	No	98.79	0.00
	Yes	1.21	
Year 5 ≥ 40% eGFR decline	No	98.78	0.00
	Yes	1.22	
Year 6 ≥ 40% eGFR decline	No	98.82	0.00
	Yes	1.18	

Abbreviations: UCLA, University of California, Los Angeles Health; PHS, Providence Health System; RUCA (rural-urban commuting area, CKD (chronic kidney disease), eGFR (estimated glomerular filtration rate), DM (diabetes mellitus), ACEI (angiotensin-converting enzyme), ARB (angiotensin receptor blockers), NSAID (non-steroidal anti-inflammatory drugs), PPI (proton pump inhibitors)

**Table 2:** Continuous variables, average value, standard deviation, and percentage of missing values. The average age of participants was 56.78 ± 17.50 years, and the average number of years followed based on eGFR labs was 3.52 ± 2.74 years. Medication prescription use is specified in days

Variable	Entry		Year 1		Year 2		Year 3		Year 4		Year 5		Year 6	
	Mean (SD)	Miss %	Mean (SD)	Miss %	Mean (SD)	Miss %	Mean (SD)	Miss %	Mean (SD)	Miss %	Mean (SD)	Miss %	Mean (SD)	Miss %
HbA1c (%)	6.57 (1.66)	79.53	6.52 (1.41)	83.16	6.48 (1.41)	84.32	6.47 (1.41)	85.81	6.47 (1.40)	87.31	6.47 (1.40)	88.89	6.50 (1.41)	90.59
UACR (mg/g)	108.98 (470.76)	95.20	102.18 (439.80)	94.57	95.23 (411.99)	94.78	95.61 (407.72)	95.20	93.80 (397.17)	95.55	95.44 (402.22)	95.55	101.34 (425.33)	96.37
UPCR (g/g)	1.47 (2.72)	99.51	1.26 (2.42)	99.49	1.18 (2.31)	99.60	1.17 (2.14)	99.63	1.18 (2.22)	99.66	1.15 (2.14)	99.63	1.15 (2.24)	99.71
SBP (mm Hg)	128.19 (16.27)	48.95	127.91 (15.33)	45.58	128.21 (15.36)	46.15	128.34 (15.24)	47.41	128.52 (15.24)	51.79	128.66 (15.24)	57.20	128.79 (15.23)	62.92
DBP (mm Hg)	74.57 (10.36)	49.03	75.40 (9.62)	45.65	75.29 (9.61)	46.21	75.45 (9.51)	47.48	75.29 (9.48)	51.85	75.16 (9.46)	57.26	75.06 (9.44)	62.96
PP (mm Hg)	53.32 (13.31)	48.91	52.48 (12.52)	45.55	52.90 (12.70)	46.11	52,87 (12.65)	47.38	53.22 (12.71)	51.76	53.48 (12.77)	57.17	53.82 (12.77)	62.89
MAP (mm Hg)	92.80 (11.15)	48.91	93.07 (10.42)	45.55	93.28 (10.38)	46.11	93.24 (10.30)	47.38	93.20 (10.27)	51.76	93.16 (10.25)	57.17	93.14 (10.21)	62.89
ACEIARB use (days)	7.10 (23.58)	0.17	33.48 (99.25)	0.36	31.94 (96.95)	0.38	33.61 (98.11)	0.53	33.69 (99.92)	0.39	29.70 (94.69)	0.28	25.42 (88.04)	0.23
NSAID use (days)	5.32 (20.18)	0.14	25.68 (84.80)	0.31	25.16 (84.60)	0.30	25.45 (84.75)	0.33	24.97 (84.67)	0.29	22.59 (81.14)	0.24	19.38 (75.53)	0.21
PPI use (days)	4.85 (19.39)	0.16	20.96 (77.14)	0.36	19.52 (74.92)	0.37	20.57 (76.25)	0.46	20.69 (77.87)	0.35	18.45 (74.15)	0.26	15.89 (69.06)	0.22
eGFR (mL/min/1.73 m^2^)	83.06 (23.97)	0.12	80.03 (24.50)	40.32	80.00 (23.82)	48.04	79.57 (23.50)	54.38	79.30 (23.22)	59.90	78.94 (22.97)	64.93	78.30 (22.77)	70.03

Abbreviations: HbA1C (hemoglobin A1c), UACR (urine albumin-creatinine ratio), UPCR (urine protein-creatinine ratio), SBP (systolic blood pressure), DBP (diastolic blood pressure), PP (pulse pressure), MAP (mean arterial pressure), eGFR (estimated glomerular filtration rate), DM (diabetes mellitus), ACEI (angiotensin-converting enzyme), ARB (angiotensin receptor blockers), NSAID (non-steroidal anti-inflammatory drugs), PPI (proton pump inhibitors)

## Evaluating the models

For each site-specific model (Fig. 3), we first test the model internally on its holdout test set and then externally using the second site’s holdout test set data (e.g., for the UCLA-learned model, we first tested on the UCLA holdout test set and then tested externally on the PHS holdout test set; and vice versa. For the UCLA+PHS model, we tested only on the UCLA+PHS holdout test set. For each model, given the available data per patient, we tested its performance for predicting ≥ 40% eGFR decline over time (i.e., in Year 1, given only data up to a single year, we determined the average precision of calculating this outcome in Years 2–6; in Year 3, given data up to Years 1–2, we determined the average precision of the model in Years 3–6). As the AUCROC metric may be misleading when training and testing on highly imbalanced datasets [25], we also calculated average precision (AP). All performance results were computed on the test set for the same site model and for the entire population during external validation. To estimate average AUCROC and AP metrics with 95% confidence intervals, 1,000 bootstrap iterations were performed, each consisting of 1,000 resampled observations.

We determined additional metrics: Brier score, precision/positive predictive value (PPV), recall/sensitivity, true positive (TP), false positive (FP), false negative (FN), and the true negative (TN) rate. To compute these metrics over time, we identified an optimal threshold for classifying a patient with ≥40% eGFR decline using the Youden statistic [26]. We conducted sensitivity analyses by trying all permutations of each variable and recorded statistically significant changes in the ≥40% eGFR decline probability every year.

The Structural Modeling, Inference, and Learning Engine (SMILE) was used to train and evaluate the DBNs [27, 28]. All models^1^ and project code^2^ are publicly available and documented for reproducibility.

## Model comparison

To assess the DBNs’ performance, we compared it with the established CKD-PC logistic regression model [17] for predicting ≥ 40% eGFR decline at Year 3 from baseline. As the CKD-PC model requires complete data (i.e., no missing values), we employed a k-nearest neighbors (KNN) imputation (k = 2) to impute missing values. We also created secondary test subsets filtered to keep only cases without missing values. Performance was computed using a bootstrap of 1,000 samples and 1,000 draws with replacement, and a proportion of 1:1 cases (balanced) of ≥40% eGFR decline and non-decliners.

Lastly, to evaluate the advantages of the over conventional ML methods, we compared its performance against four common ML classifiers: CatBoost, logistic regression, random forest, and XGBoost. The models were trained to predict a ≥ 40% decline in eGFR over 1 year and tested over a 6-year longitudinal horizon. Model performance was computed using a bootstrap of 1,000 samples and 1,000 draws with replacement, and a stratified proportion of cases of ≥40% eGFR decline and non-decliners over each year, similar to the DBNs.

## Results

Demographic and clinical characteristics of the study sample are provided in Tables 1 and 2, including missingness rates. Most of the population was White non-Latino (68%), with 89% residing in urban areas. In terms of the outcome, a total of 6.49% of patients had at least one eGFR measurement with ≥40% decline in the follow-up period. HbA1C, UACR, and urine protein-to-creatinine ratio (UPCR) had the highest missing values, with rangees of 79.53–90.59%, 95.2–96.37%, and 99.51–99.71%, respectively, over the 6-year period (Table 2). eGFR measurement decreased with population age and reached a maximum missingness rate of 70.03%.

Our longitudinal analysis of CURE-CKD revealed that a ≥ 40% decline in eGFR was frequently a non-terminal, fluctuating event in real-world clinical practice, often reflecting the resolution of acute kidney injury (AKI) or acute kidney disease (AKD) episodes rather than immediate, irreversible progression to end-stage kidney disease. We observed that approximately 23% of patients who experienced recurrent decline events also demonstrated significant eGFR upswings (>20%), underscoring the dynamic nature of kidney function over a longer-term observational horizon. The results of this analysis are depicted in section 1.9 of Supplemental Materials.

## Model relationships and sensitivity analysis

**Fig. 5:**
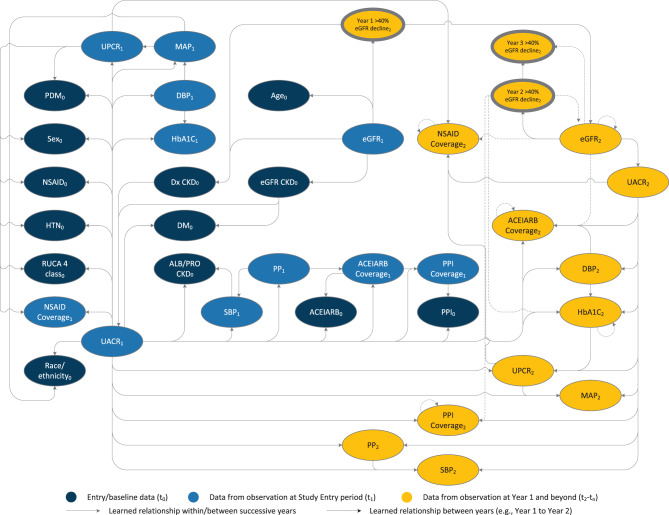
Abbreviated UCLA-PHS dynamic Bayesian network, showing the relationships between study entry and initial observations. Additional relationships to follow-up years are noted. Solid arrows represent conditional dependence, and dashed arrows represent temporal dependence. Abbreviations: UCLA, University of California, Los Angeles Health; PHS, Providence Health System; RUCA (rural-urban commuting area), CKD (chronic kidney disease), HbA1C (hemoglobin A1c), UACR (urine albumin-creatinine ratio), UPCR (urine protein-creatinine ratio), SBP (systolic blood pressure), DBP (diastolic blood pressure), PP (pulse pressure), MAP (mean arterial pressure), eGFR (estimated glomerular filtration rate), DM (diabetes mellitus), ACEI (angiotensin-converting enzyme), ARB (angiotensin receptor blockers), NSAID (non-steroidal anti-inflammatory drugs), PPI (proton pump inhibitors)

**Fig. 6:**
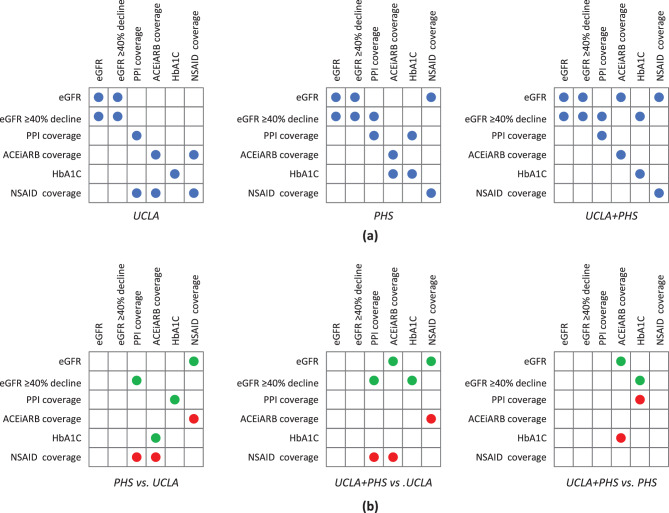
Matrix representations of the DBN structures, looking at relationships over time. Row-to-column indicates the direction of an edge, with the directionality representing statistical dependence (e.g., variable a is the parent of variable B). (**a**) Temporal connections between successive years for each site, with each blue dot representing a connection over time; (**b**) Comparing how the time-based relationships differ between sites, with a red dot representing a missing edge and a green dot representing a new edge. Abbreviations: UCLA, University of California, Los Angeles Health; PHS, Providence Health System; RUCA (rural-urban commuting area), CKD (chronic kidney disease), HbA1C (hemoglobin A1c), UACR (urine albumin-creatinine ratio), UPCR (urine protein-creatinine ratio), SBP (systolic blood pressure), DBP (diastolic blood pressure), PP (pulse pressure), MAP (mean arterial pressure), eGFR (estimated glomerular filtration rate), DM (diabetes mellitus), ACEI (angiotensin-converting enzyme), ARB (angiotensin receptor blockers), NSAID (non-steroidal anti-inflammatory drugs), PPI (proton pump inhibitors)

**Fig. 7:**
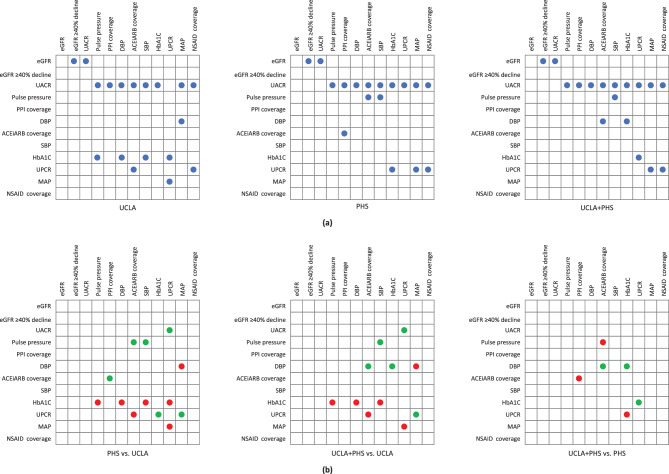
Matrix representation of the DBNs, looking at relationships between observations within the same time period. Row-to-column indicates the direction of an edge, with the directionality representing statistical dependence (e.g., variable a is the parent of variable B). (**a**) Relationships for each site. (**b**) Again, there are differences between the sites, with a red dot representing a missing edge and a green dot representing a new edge. Abbreviations: UCLA, University of California, Los Angeles Health; PHS, Providence Health System; RUCA (rural-urban commuting area), CKD (chronic kidney disease), HbA1C (hemoglobin A1c), UACR (urine albumin-creatinine ratio), UPCR (urine protein-creatinine ratio), SBP (systolic blood pressure), DBP (diastolic blood pressure), PP (pulse pressure), MAP (mean arterial pressure), eGFR (estimated glomerular filtration rate), DM (diabetes mellitus), ACEI (angiotensin-converting enzyme), ARB (angiotensin receptor blockers), NSAID (non-steroidal anti-inflammatory drugs), PPI (proton pump inhibitors)

Figure 5 shows the learned DBN for UCLA+PHS. To extract the temporal structure (i.e., the temporal variables tracked during the CURE-CKD registry follow-up period) characteristics, we analyzed and compared the structures between the sites. The models’ structures consisted of two parts: 1) edges between variables over time (e.g., between the current and past year); and 2) edges between observations at the same time/year. Figures 6 and 7 describe this graphical structure in terms of matrices that depict the edges (statistical dependence, from rows to columns) between variables. Supplemental Materials Section 1.5 contains matrices of the non-temporal structure (i.e., variables of study entry and entry period) of the DBNs for each site. The diagonal of Fig. 6a, for example, indicates that for all sites, the current value of a variable depended on the previous year’s values. Figure 6b shows the differences in temporal edges between variables for each site. Here, our temporal analysis revealed that edges towards outcomes (e.g., eGFR decline ≥ 40%) were consistent throughout all sites’ models. Notably, most differences arose because of medication and HbA1c variables with high missing value rates. Figure 7a demonstrates that the most “dependent” variables within a year’s values were UACR, UPCR, and blood pressure, although the directionality of dependency changed between sites for some variables (Fig. 7b). Likewise, most differences were between variables with high missing values, including medication, blood pressure, and HbA1c.

Applying sensitivity analysis to key DBN variables indicated that eGFR, UACR, and pulse pressure were the most influential on the target outcome. In this light, model performance for patients with albuminuria and/or proteinuria (UACR > 30 mg/g and/or UPCR > 150 mg/g) and established CKD was higher than the rest of the population, indicating that the model captures phenotypes exhibited by these patients with high risk of losing kidney function.

## Model performance

**Fig. 8:**
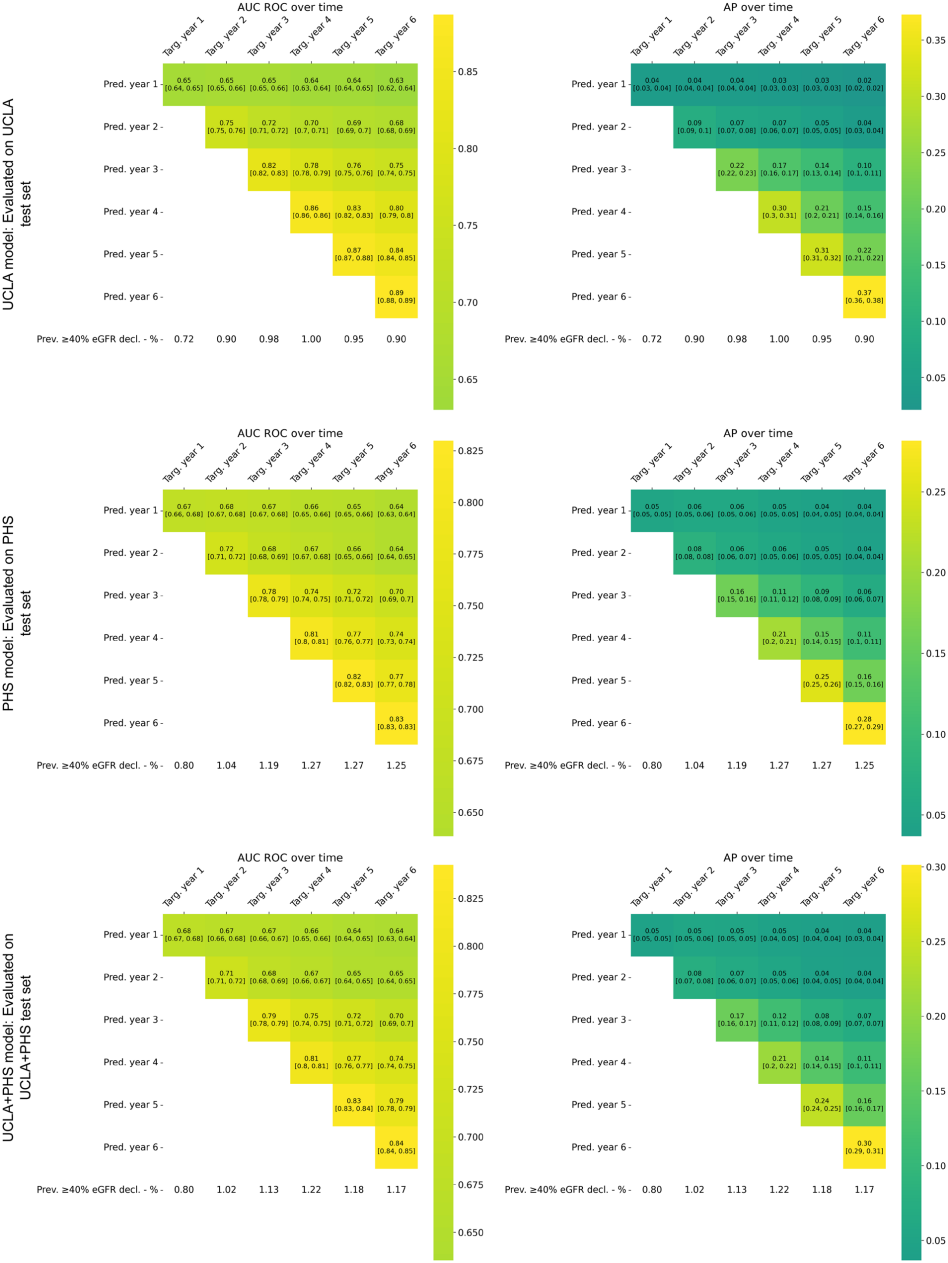
DBNs AUCROC and AP over time on the hold-out test set of each site. Pred. Year: represents the model’s outcome prediction for a year using observations before that year. Targ Year: represents the ground truth of ≥40% eGFR decliners

The DBNs performance improved significantly over time, peaking at Year 6 (see Fig. 8) to identify ≥ 40% eGFR decline across the two health systems and models. Specifically, the AUCROC increased from 0.65–0.68 to 0.83–0.89 over time (Year 1 to Year 6), while AP improved from 0.04–0.05 to 0.28–0.37. This point emphasizes the importance of integrating past and current observations to improve the prediction of eGFR trajectories when such information is available. Table 3 shows the models’ performance at Year 6 when stratifying the population by comorbidities, race, and ethnicity at baseline. When limiting the model to cases of CKD at baseline from high UACR/UPCR, the models performance improved with an AP of 0.49, 0.46, and 0.45, and AUCROC of 0.90, 0.86, and 0.86 for the UCLA, PHS, and UCLA-PHS models, respectively.

**Table 3:** Stratified population performance metrics for each model with 95% confidence intervals at Year 6

	UCLA		PHS		UCLA+PHS	
	AUCROC	AP	AUCROC	AP	AUCROC	AP
Whole population	0.89 (0.88–0.89)	0.37 (0.36–0.38)	0.83 (0.83–0.83)	0.28 (0.27–0.29)	0.84 (0.84–0.85)	0.30 (0.29–0.31)
Albuminuria/ Proteinuria	0.90 (0.90–0.90)	0.49 (0.49–0.50)	0.86 (0.86–0.86)	0.46 (0.46–0.46)	0.86 (0.86,0.86)	0.45 (0.44–0.45)
At-risk of CKD	0.89 (0.88–0.89)	0.38 (0.36–0.39)	0.81 (0.81–0.82)	0.26 (0.25–0.27)	0.82 (0.82–0.83)	0.28 (0.27–0.28)
CKD	0.82 (0.81–0.82)	0.34 (0.33–0.35)	0.84 (0.83–0.84)	0.33 (0.33–0.34)	0.83 (0.83–0.84)	0.33 (0.33–0.34)
CKD from Diagnosis code	0.84 (0.84–0.84)	0.37 (0.37–0.38)	0.89 (0.89–0.89)	0.39 (0.39–0.40)	0.88 (0.88–0.89)	0.41 (0.40–0.41)
eGFR lab CKD	0.79 (0.79–0.80)	0.32 (0.31–0.33)	0.84 (0.84–0.85)	0.34 (0.34–0.35)	0.83 (0.83–0.84)	0.33 (0.33–0.34)
Stage 1 CKD	0.88 (0.87–0.88)	0.43 (0.42–0.44)	0.76 (0.76–0.77)	0.22 (0.21–0.23)	0.79 (0.78–0.80)	0.27 (0.26–0.28)
Stage 2 CKD	0.91 (0.90–0.92)	0.38 (0.37–0.39)	0.85 (0.85–0.85)	0.30 (0.29–0.30)	0.85 (0.84–0.85)	0.29 (0.29–0.30)
Stage 3a CKD	0.88 (0.88–0.88)	0.37 (0.37–0.38)	0.82 (0.82–0.82)	0.31 (0.30–0.32)	0.82 (0.82–0.82)	0.31 (0.30–0.31)
Stage 3b CKD	0.70 (0.70–0.71)	0.33 (0.32–0.33)	0.86 (0.86–0.87)	0.35 (0.34–0.35)	0.84 (0.84–0.85)	0.34 (0.34–0.35)
Stage 4 CKD	0.73 (0.72–0.73)	0.28 (0.28–0.29)	0.88 (0.88–0.88)	0.43 (0.43–0.43)	0.84 (0.84–0.84)	0.38 (0.38–0.39)
Stage 5 CKD	0.53 (0.53–0.54)	0.08 (0.08–0.08)	0.76 (0.76–0.76)	0.17 (0.17–0.18)	0.80 (0.80–0.81)	0.20 (0.20–0.20)
Non-Hispanic	0.88 (0.88–0.89)	0.36 (0.35–0.37)	0.83 (0.83–0.84)	0.29 (0.28–0.30)	0.83 (0.83–0.84)	0.28 (0.28–0.29)
Hispanic	0.91 (0.91–0.92)	0.44 (0.43–0.44)	0.85 (0.85–0.86)	0.31 (0.30–0.32)	0.87 (0.87–0.87)	0.39 (0.38–0.40)
Non-Black	0.89 (0.89–0.89)	0.37 (0.36–0.38)	0.83 (0.82–0.83)	0.28 (0.27–0.29)	0.83 (0.83–0.84)	0.29 (0.28–0.30)
Black	0.85 (0.85–0.86)	0.33 (0.32–0.34)	0.86 (0.86–0.86)	0.38 (0.37–0.39)	0.86 (0.86–0.87)	0.35 (0.35–0.36)
Non-Native American	0.89 (0.88–0.89)	0.37 (0.36–0.38)	0.83 (0.82–0.83)	0.29 (0.28–0.30)	0.84 (0.84–0.85)	0.31 (0.30–0.32)
Native American	0.88 (0.88–0.88)	0.29 (0.29–0.30)	0.85 (0.85–0.86)	0.33 (0.32–0.34)	0.78 (0.78–0.79)	0.28 (0.27–0.29)
Non-White	0.91 (0.90–0.91)	0.40 (0.39–0.41)	0.85 (0.85–0.85)	0.35 (0.34–0.36)	0.86 (0.85–0.86)	0.35 (0.34–0.36)
White	0.87 (0.87–0.88)	0.34 (0.33–0.35)	0.82 (0.82–0.83)	0.27 (0.26,0.28)	0.83 (0.83–0.84)	0.27 (0.27–0.28)
Male	0.88 (0.88–0.89)	0.38 (0.37–0.39)	0.83 (0.82–0.83)	0.30 (0.29–0.30)	0.85 (0.84–0.85)	0.31 (0.31–0.32)
Female	0.89 (0.89–0.90)	0.35 (0.34–0.36)	0.83 (0.83–0.84)	0.29 (0.28–0.30)	0.83 (0.83–0.83)	0.29 (0.28–0.30)

Abbreviations: AUCROC (area under the curve of the receiver operating characteristic), AP (average precision), CKD (chronic kidney disease), eGFR (estimated glomerular filtration rate)

**Fig. 9:**
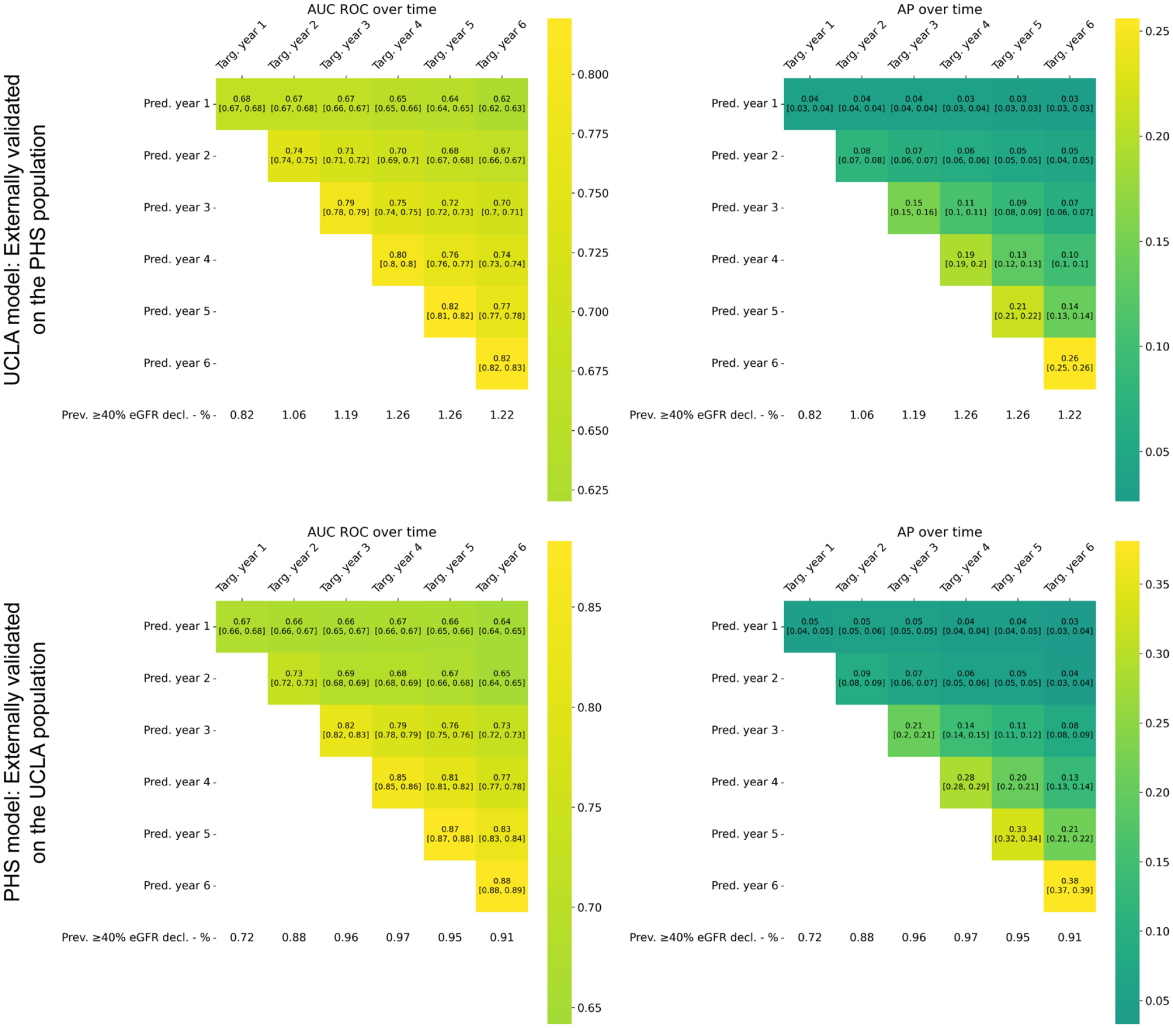
DBNs AUCROC and AP over time on external validation sets. Pred. Year: represents the model’s outcome prediction for a year using observations before that year. Targ. Year: represents the ground truth of ≥40% eGFR decliners

Figure 9 summarizes performance over time regarding external validation of the models. The UCLA model performance on the PHS population decreased AP over time (per the diagonal of Fig. 9) as compared with the performance of the PHS model on the PHS holdout test set population (per the diagonal of Fig. 8). However, the PHS model AP on the UCLA population improved over time as compared to the UCLA model on the UCLA test set population (diagonals of Figs. 8 and 9), with APs of 0.38 and 0.36 at Year 6, respectively. The PHS model demonstrated more significant improvement compared to the UCLA model in terms of AUCROC and AP for the UCLA population, in established CKD subgroups such as CKD from diagnosis codes or eGFR, as shown in Tables 3 and 4. Stratifying both the at-risk and CKD population by CKD stage at baseline (i.e., CKD stages 1–5, which represent kidney function stages measured by KDIGO eGFR categories; with increasing stage corresponding to an increased loss of kidney function), we observed the model performance was more stable and improved across CKD stage subgroups with DBN models trained on a larger dataset (PHS and UCLA+PHS in Table 3). In Table 4, the PHS model exhibited significantly improved performance across CKD stage subgroups in the UCLA population compared to the UCLA model presented in Table 3. The DBNs additional performance metrics and validation performance metrics for each site are shown in the Supplemental Materials, Section 1.4.2 and Section 1.6, respectively.

**Table 4:** Stratified population performance metrics for each model tested on the other health system, with 95% confidence intervals at Year 6

	UCLA model on PHS population		PHS model on UCLA population	
	AUCROC	AP	AUCROC	AP
Whole population	0.82 (0.82–0.83)	0.26 (0.25–0.26)	0.88 (0.88–0.89)	0.38 (0.37–0.39)
Albuminuria/ proteinuria	0.86 (0.85–0.86)	0.42 (0.42–0.42)	0.89 (0.89–0.89)	0.46 (0.46–0.47)
At-risk of CKD	0.81 (0.81–0.82)	0.23 (0.22–0.24)	0.87 (0.86–0.88)	0.39 (0.37–0.40)
CKD	0.81 (0.81–0.82)	0.30 (0.29–0.31)	0.86 (0.86–0.86)	0.38 (0.37–0.39)
CKD from diagnosis code	0.86 (0.86–0.87)	0.36 (0.36–0.37)	0.87 (0.87–0.87)	0.43 (0.43–0.44)
eGFR lab CKD	0.81 (0.81–0.81)	0.29 (0.29–0.30)	0.87 (0.86–0.87)	0.38 (0.37–0.39)
Stage 1 CKD	0.78 (0.78–0.79)	0.23 (0.22–0.24)	0.88 (0.88–0.89)	0.38 (0.37–0.39)
Stage 2 CKD	0.84 (0.83–0.84)	0.25 (0.25–0.26)	0.89 (0.88–0.89)	0.39 (0.38–0.40)
Stage 3a CKD	0.83 (0.82–0.83)	0.28 (0.28–0.29)	0.88 (0.87–0.88)	0.39 (0.38–0.40)
Stage 3b CKD	0.75 (0.75–0.76)	0.28 (0.28–0.29)	0.88 (0.87–0.88)	0.39 (0.38–0.40)
Stage 4 CKD	0.83 (0.83–0.83)	0.38 (0.37–0.38)	0.86 (0.86–0.87)	0.34 (0.33–0.35)
Stage 5 CKD	0.79 (0.79–0.90)	0.22 (0.21–0.22)	0.83 (0.82–0.83)	0.37 (0.36–0.38)
Non-Hispanic	0.83 (0.83–0.83)	0.26 (0.25–0.27)	0.88 (0.88–0.88)	0.37 (0.36–0.38)
Hispanic	0.86 (0.85–0.86)	0.28 (0.28–0.29)	0.90 (0.89–0.90)	0.46 (0.45–0.47)
Non-Black	0.82 (0.82–0.83)	0.25 (0.25–0.26)	0.88 (0.87–0.88)	0.39 (0.38–0.40)
Black	0.85 (0.85–0.86)	0.33 (0.32–0.33)	0.89 (0.88–0.89)	0.42 (0.41–0.42)
Non-Native American	0.83 (0.82–0.83)	0.26 (0.25–0.27)	0.88 (0.88–0.89)	0.38 (0.37–0.39)
Native American	0.81 (0.80–0.81)	0.28 (0.27–0.29)	0.89 (0.89–0.90)	0.41 (0.40–0.42)
Non-White	0.85 (0.84–0.85)	0.31 (0.30–0.32)	0.90 (0.89–0.90)	0.42 (0.41–0.43)
White	0.82 (0.82–0.83)	0.25 (0.24–0.26)	0.87 (0.86–0.87)	0.34 (0.33–0.35)
Male	0.84 (0.83–0.84)	0.27 (0.26–0.28)	0.87 (0.87–0.88)	0.37 (0.36–0.38)
Female	0.82 (0.82–0.83)	0.26 (0.25–0.26)	0.88 (0.88–0.89)	0.39 (0.38–0.40)

Abbreviations: AUCROC (area under the curve of the receiver operating characteristic), AP (average precision), CKD (chronic kidney disease), eGFR (estimated glomerular filtration rate)

Subgroup analyses involving racial and ethnic minority groups showed higher performance over time and overall better performance, at Year 6, than comparison race groups (Table 4). The UCLA Hispanic subgroup had the highest AP and AUCROC compared to all other racial and ethnic groups at Year 6, at 0.40 and 0.91, respectively. The PHS Black subgroup had the highest AP and AUCROC, compared to all other race and ethnic groups, at Year 6 of 0.38 and 0.86, respectively. The PHS Native American or Alaska Native subgroup showed the second-highest AP and AUCROC at Year 6, at 0.33 and 0.85, respectively. A comparison between male and female subgroups did not show significant differences within and across health systems.

## Comparing performance to the CKD-PC model with and without missing data

CKD-PC performed poorly on a dataset with missing values (AUCROC 0.53–0.55, AP 53–0.54) relative to the DBN models (AUCROC 0.65–0.68, AP 0.64–0.68) (Table 5). CKD-PC outperformed the DBNs on the filtered sets (i.e., with complete data) with AUCROC 69–0.71 and AP 0.69–0.72 and AUCROC 65–0.67 and AP 0.63–0.68, respectively. Filtered datasets (without cases of missing values) correspond to a large reduction in the number of instances in the test set (>95% of the whole test set). In contrast, performance of the DBNs was unaffected by missing data.

**Table 5:** Comparison of CKD-PC model on a stratified balanced population at Year 3. AUCROC and AP performance metrics for each model are shown with 95% confidence intervals. The sample size when using imputation for UCLA, PHS, and UCLA+PHS test set was 66,639, 383,523, and 450,162, respectively. The sample size when filtering out cases with missing values in any of the variables used by the CKD-PC model (age, sex, eGFR, UACR, and diabetes) for UCLA, PHS, and UCLA+PHS test set, 3,933, 17,618, and 21,605, respectively

	UCLA		PHS		UCLA+PHS	
	AUCROC	AP	AUCROC	AP	AUCROC	AP
CKD-PC (kNN imputation)	0.537 (0.536–0.538)	0.541 (0.540–0.542)	0.544 (0.543–0.546)	0.530 (0.529–0.531)	0.550 (0.549–0.551)	0.539 (0.538,0.540)
CKD-PC (complete dataset)	0.694 (0.693–0.695)	0.695 (0.693–0.696)	0.709 (0.708–0.711)	0.716 (0.715–0.717)	0.700 (0.698–0.701)	0.703 (0.702–0.704)
UCLA DBN	0.655 (0.639–0.656)	0.639 (0.638–0.640)	-	-	-	-
UCLA DBN (complete dataset)	0.648 (0.647–0.649)	0.632 (0.631–0.633)	-	-	-	-
PHS DBN	-	-	0.675 (0.673–0.676)	0.678 (0.677–0.679)	-	-
PHS DBN (complete dataset)	-	-	0.674 (0.673–0.675)	0.676 (0.675–0.677)	-	-
UCLA+PHS DBN	-	-	-	-	0.670 (0.669–0.671)	0.674 (0.673–0.676)
UCLA+PHS DBN (complete dataset)	-	-	-	-	0.670 (0.669–0.671)	0.675 (0.673–0.676)

## Comparing DBN performance to other machine learning models

A distinct “crossover” effect was observed in the DBN model performance. In short-term predictions (Year 1), static ML models performed competitively, with XGBoost achieving the highest initial AUCROC (0.75) and AP (0.08). But as the prediction window was extended over time, and the clinical data became increasingly sparse (reaching 70% missingness by Year 6), the performance of the static models plateaued or declined. In contrast, the DBN demonstrated superior scalability and robustness. By modeling the temporal dependencies and functional fluctuations (upswings and downswings) of eGFR, the DBN performance increased over time. By Year 6, the DBN achieved AUCROC of 0.84 and AP of 0.30, outperforming all baseline ML models. Each model performance over time is shown in Supplemental Materials, Section 1.7.

## Discussion

We leveraged two large healthcare systems EHRs to build ML models to identify patients with ≥40% eGFR decline over six years. Importantly, these DBN models were trained and tested on the full dataset with missing data, an inherent problem with all EHRs (e.g., MIMIC-III, a health-related dataset comprising over 40,000 patients, has missing value rates as high as 96%) [29]. Given the ability of DBNs to handle missing data, the models simulated missing values for all patient variables over a 6-year horizon to predict the risk of annual ≥ 40% eGFR decline. Notably, most observational datasets used in model building contain missing values that are missing not at random or missing at random, and as such the models are subject to interpretation [30–32]. Thus, unlike other ML models that require complete data for training and testing (e.g., a logistic regression model), the DBN can utilize available EHR datasets and provide a more pragmatic clinical decision support tool that does not require *all* data to be available to provide predictive insights. Despite the high rate of missingness observed in the CURE-CKD cohort, our model’ performance improved as data accrued, suggesting that the models were also learning key relationships that drive loss of kidney function. Markedly, our models exhibited AUCROC of 0.83–0.89 and average precision of 0.28–0.37 (Year 6) on a holdout test set and in testing within alternative healthcare systems, suggesting this approach is feasible and generalizable.

Our choice in using DBNs was enhanced by their inherent explainability of results: unlike other contemporary ML techniques (e.g., deep learning), explanations can be automatically derived and presented to users without secondary interpretation methods [33]. Upon analyzing the temporal structure of each health system (Figs. 6 and 7), we observed minimal differences across sites, with most variations occurring in medication use, UPCR, HbA1C, and blood pressure-related variables. These represent the variables with the highest missing values (Table 2). Obtaining these laboratory values is contingent on the discretion of a clinician and may be biased by confounders such as CKD awareness or access to care. An estimate or the actual value of these variables provides an opportunity to improve monitoring of kidney function over time. The DBNs can predict ≥ 40% eGFR decline several years in advance and become more accurate as observations accrue (Figs. 8 and 9), suggesting that the derived temporal structure can reliably simulate missing values and improve outcome predictions.

In developing health system-specific models and with relevant external validation, we observed differences in performance. When evaluating the UCLA model, we observed that it did not generalize as well as the converse (i.e., the PHS model tested on the UCLA population). This finding suggested we needed to train and move towards bigger datasets, as the number of individuals from PHS was markedly larger than that from UCLA. We thus also developed the UCLA+PHS model, with the resultant graphical structure showing that their combination yielded a structure similar to that of each separate population.

Our sensitivity analysis highlighted variables such as eGFR, UACR/UPCR and pulse pressure as the most influential to outcomes. In this light, model performance for patients with UACR/UPCR and a CKD diagnosis was higher than the rest of the population, indicating that the model captures phenotypes exhibited by these patients at-risk of kidney decline. This finding was also seen in patients belonging to different races and racial ethnic groups (Hispanic, Black, and Native Americans) who continue to experience health disparities related to treatment and outcomes [34, 35].

This study utilizes real-world clinical data, which, while reflective of routine healthcare settings, commonly contain a high proportion of missing values, a well-known challenge in observational datasets [36, 37]. We compared the DBNs with the published CKD-PC model. Despite the good performance of the CKD-PC model on a subset of our cohort test sets with complete data (i.e., no missing values), there were limitations. First, many models, similar to CKD-PC, require *all* variables available to calculate the risk of losing kidney function as missing data is not readily handled, and in some cases, the model may involve an infrequently captured type of data, limiting translation into real-world clinical settings. This issue is highlighted when comparing our DBN models to the CKD-PC model in scenarios of complete and missing data. The CKD-PC cannot be used on cases with missing values, which represent > 93% of all subjects identified in the CURE-CKD cohort: when applied to the entire dataset with imputation, it performed close to random; the DBNs’ performance, on the other hand, remained unaffected by missing values. When used only on cases with the five variables of the CKD-PC, and with all laboratory values present, the equation outperformed our DBNs by <0.05 AUCROC. This comparison highlights the gap in predicting eGFR decline in the presence of missing data, a reality of routine clinical care. Second, these traditional models are static, using only a single time point (i.e., most recent observations) to assess future risk rather than on longitudinal observations in a given individual. While static models are more straightforward to understand and implement, longitudinal changes can better help predict long-term outcomes and uncover potential points for influencing kidney function, as shown through our comparison with traditional ML models. This was also enhanced by our analysis of the DBN structure, which highlighted a well-known intuition (the Markov assumption) that current variables’ values are dependent on their previous values (e.g., annual change in eGFR) and therefore predictive in longer-term trajectories (i.e., the diagonal of Fig. 6a).

Our objective in the current study was to provide insights into the factors that influence substantial eGFR decline over time and an early indicator of who may be at-risk long before kidney failure develops. Still, we recognize that our current modeling work has limitations. For instance, the learned DBNs, although relatively consistent across sites, might be incomplete or inaccurate in variables with high missing values. Moreover, our DBNs are discrete, with all categorical variables for explainability and simplicity. It is likely that continuous values for variables, such as age or eGFR, can improve model performance. Similarly, our prediction of ≥40% eGFR decline is annual (i.e., discrete in time), and a continuous-time DBN would further improve the time of eGFR decline prediction, albeit given further computational complexity.

This inherent variability in clinical trajectories explains the performance “crossover” observed in our comparative experiments, between traditional ML models and the UCLA+PHS DBN. ML models rely on static snapshots of data and struggle to maintain predictive performance as clinical records become increasingly sparse and erratic. Conversely, the DBN leveraged its probabilistic framework to model temporal dependencies and inferred missing values, resulting in improved long-term performance for this predictive task. These findings suggest that dynamic modeling is a reliable modeling approach for populations with or at risk of CKD.

In conclusion, the DBNs we constructed using real-world EHR data can effectively handle substantial missing data over a 6-year horizon and predict the risk of ≥40% eGFR decline annually. Model performance improved even though the rate of missing values increased over time, suggesting an accurate simulation of missing values as data accrued. Future work will include using our methods to explore newer, kidney-protective therapies for CKD, such as sodium-glucose cotransporter-2 (SGLT-2) inhibitors, and other clinical variables, including social determinants of health. We also expect to expand the scope of CURE-CKD to other populations representative of the US, creating a deeper understanding of the issues driving loss of kidney function across different groups and learning systems that can inform clinicians sufficiently early to consider kidney-protective interventions.

## Electronic supplementary material

Below is the link to the electronic supplementary material.

Supplementary Material 1

## Data Availability

The data that support the findings of this study are available upon reasonable request from the corresponding author, Dr. Panayiotis Petousis, and the CURE-CKD team. The datasets analyzed consist of aggregated health care data, observational records, research protocols, and statistical analysis plans. The data are not publicly available because they constitute limited datasets containing protected health information from two US health systems (Providence and UCLA Health). Consequently, access is subject to privacy and ethical restrictions and would require a formal Data Use Agreement (DUA).

## Abbreviations

ACEI: Angiotensin-Converting Enzyme Inhibitor
AKI: Acute Kidney Injury
AP: Average Precision
ARB: Angiotensin Receptor Blocker
AUCROC: Area Under the Receiver Operating Characteristic Curve
BN: Bayesian Network
CKD: Chronic Kidney Disease
CKD-PC: Chronic Kidney Disease Prognosis Consortium
CURE-CKD: Center for Kidney Disease Research, Education, and Hope
DBN: Dynamic Bayesian Network
DBP: Diastolic Blood Pressure
DM: Diabetes Mellitus
Dx: Diagnosis
eGFR: Estimated Glomerular Filtration Rate
eGFR CKD: Chronic Kidney Disease identified from estimated GFR
EM: Expectation-Maximization
FN: False Negative
FP: False Positive
HbA1c / HbA1C: Hemoglobin A1c
ICD: International Classification of Diseases
IRB: Institutional Review Board
KNN: K-Nearest Neighbors
KFRE: Kidney Failure Risk Equation
MAP: Mean Arterial Pressure
ML: Machine Learning
NSAID: Non-Steroidal Anti-Inflammatory Drug
PHS: Providence Health System
PP: Pulse Pressure
PPI: Proton Pump Inhibitor
PPV: Positive Predictive Value
RAUS: Ranking Approaches for Unknown Structures
RF: Random Forest
RUCA: Rural-Urban Commuting Area
SBP: Systolic Blood Pressure
SGLT-2: Sodium-Glucose Cotransporter-2
SMILE: Structural Modeling, Inference, and Learning Engine
TN: True Negative
TP: True Positive
UACR: Urine Albumin-Creatinine Ratio
UCLA: University of California, Los Angeles Health
UPCR: Urine Protein-Creatinine Ratio
US: United States

## Footnotes

1 https://zenodo.org/uploads/13787736

2 https://github.com/uclamii/DBN_simulation_modeling
