# Supplementary material for: Dynamic Bayesian networks to predict loss of kidney function: a cross-institution use case in a large cohort with or at-risk of CKD
Source: BMC Med Inform Decis Mak. 2026 May 20;26:264. doi: 10.1186/s12911-026-03570-6 (PMC13366859; doi:10.1186/s12911-026-03570-6)
Supplement: Supplementary file 1 — Supplementary Material 1 [file 12911_2026_3570_MOESM1_ESM.docx]

# Supplemental Materials

## 1.1 CURE-CKD Registry Inclusion and Exclusion Criteria

*CURE-CKD Repository*. Patients are identified with candidate conditions in each health system (University of California, Los Angeles Health System and Providence Health System), with demographic and clinical data extracted from the electronic health record (EHR). Fifteen clinical rules qualified a patient for incorporation into the overarching CURE-CKD cohort:

1. Laboratory record indicating an estimated glomerular filtration rate (eGFR) less than or equal to 60 ml/min/1.73 m^2^.
2. Any encounter with a diagnosis code International Classification of Diseases 9/10 (ICD-9/10) indicating prediabetes or diabetes mellitus (DM).
3. Laboratory record indicating fasting blood glucose between 100-125 mg/dL.
4. Laboratory record indicating fasting blood glucose greater than or equal to 126 mg/dL.
5. Any encounter with a diagnosis code (ICD09 or ICD10) indicating DM.
6. An order for medications prescribed to treat DM (sulfonylurea, thiazolidinedione, meglitinide, amylin analog, glucagon-like peptide-1 agonist, dipeptidyl peptidase 4 inhibitor, sodium-glucose cotransporter-2 inhibitor, alpha-glucosidase, biguanide, insulin or dopamine receptor agonists).
7. Laboratory record indicating random blood glucose between 140-199 mg/dL.
8. Any encounter with a diagnosis code (ICD-9/10) indicating chronic kidney disease.
9. Laboratory record indicating random blood glucose greater than or equal to 200 mg/dL.
10. Laboratory record indicating hemoglobin A1c greater than or equal to 6.5%.
11. Laboratory record indicating hemoglobin A1c greater than or equal to 5.7% and less than 6.5%.
12. A systolic blood pressure (SBP) on any encounter greater than or equal to 140 mm Hg or diastolic blood pressure (DBP) on any encounter greater than or equal to 90 mm Hg.
13. Any encounter with a diagnosis code (ICD-9/10) indicating hypertension.
14. Laboratory record indicating urine albumin to creatinine ratio (UACR) greater than 30 mg/g.
15. Laboratory record indicating urine protein to creatinine ratio (UPCR) greater than 150 mg/g.

*Clinical thresholds and missing values.* These thresholds were provided by clinicians to further refine and filter the dataset:

- Replace all 0 HbA1c values as missing.
- Replace urine UACR below 0.06 and above 10,000 as missing.
- Replace urine UPCR above 10,000 or below 0.1 as missing.
- Replace SBP readings above 200 mm Hg and below 60 mm Hg as missing.
- Replace DBP readings above 110 mm Hg and below 30 mm Hg as missing.
- Replace eGFR race and no-race adjusted readings above 185 ml/min/1.73 m^2^ and below 5 ml/min/1.73 m^2^ as missing.

In addition, all subjects included in this specific study had at least 1 year of follow-up to enable evaluation of the prediction task.

## 1.2 Data Discretization

In this work, we have built discrete DBNs that require input variables to be discretized (qualitative and/or categorical variables). We employ minimum description length (MDL) discretization via the Orange package to automatically create discrete bins on continuous variables. The MDL of an object represents the minimum number of bits required to uniquely specify an object in the world of objects. MDL seeks to find an optimal set of cut points that divide a continuous variable into distinct intervals or bins. MDL discretization typically involves iteratively evaluating different cut points and assessing the resulting data compression. MDL discretization is a supervised algorithm that recursively decides whether to further split or accept a given partition of continuous data based on the MDL Principle criterion. The MDLP criterion uses information gain to accept or reject an attribute's discretization.

## 1.3 Defining the Dynamic Bayesian Network Topology

Current network topology learning algorithms require ordering variable feature importance to suggest potential graphs. To this end, we used the Ranking Approaches for Unknown Structures (RAUS) to learn the model topology automatically and perform basic checks on the soundness of the statistical associations. RAUS learns unknown structures via competing well-established filter/rank methods (chi-squared, Cramer's V, information gain) for sorting input variables to greedy search methods, which are a lower cost and scalable approach for use on large datasets in comparison to alternatives, such as exhaustive search or Markov Chain Monte Carlo (MCMC) methods. In this work, RAUS uses each site’s training set to learn a set of topologies and uses the validation set to identify the best topology by building a belief network or DBN. We used a mixture of (unknown) structure learning experts (MoslE) approach from RAUS, to learn the contemporals, the conditional relationships within a given observation period (e.g., dependencies of variables within Year 1), and the temporally dependent relationships projected forward between observation periods (e.g., dependencies of variables in Year 2 from Year 1 or baseline observations). Here, the contemporal substructure represents a directed acyclic graph (DAG) over CURE-CKD study entry and entry period variables. Amazon Web Services (AWS) was used to run RAUS across multiple scenarios, including considering models with and without race-related variables to assess potential biasing. Identifying multiple substructures using RAUS on a large dataset such as CURE-CKD is computationally expensive. Given RAUS's modularity, it can be scaled across cluster cloud services. In this work, we used Amazon Web Services (AWS) to identify the optimal topology, with each run taking 5-14 days on four parallel c5.24xlarge virtual machine (VM) instances.

## 1.4 DBNs Supplemental Evaluation Metrics

### 1.4.1 Optimal Annual Probability Thresholds

Using the validation set per site, we iterated over the probability space in steps of 0.01 and computed the Youden statistic. The threshold value with the highest statistic was selected as the optimal threshold for each time point of the 6-year horizon.

**Supplemental Table 1:** Optimal probability thresholds for each model over time.

|  | Optimal probability threshold | | |
| --- | --- | --- | --- |
| Model | **UCLA** | **PHS** | **UCLA+PHS** |
| Year 1 | 0.12 | 0.10 | 0.10 |
| Year 2 | 0.17 | 0.17 | 0.18 |
| Year 3 | 0.14 | 0.18 | 0.17 |
| Year 4 | 0.19 | 0.18 | 0.19 |
| Year 5 | 0.13 | 0.16 | 0.15 |
| Year 6 | 0.02 | 0.03 | 0.03 |

### 1.4.2 Additional performance metrics

DBN models’ performance with and without 95% confidence intervals (CIs) is accompanied by a zip file^[[1]](#footnote-1)^ depicting precision (PPV, positive predictive value), recall (sensitivity), specificity, F1-score, AUCROC, AP, FN (false negative), TP (true positive), FP (false positive), TN (true negative), and Brier scores. The metrics are illustrated for the complete test sets and external validation sets, as well as metrics for a stratified (in terms of outcome) bootstrap results analysis and a balanced (in terms of outcome) bootstrap results analysis.

## DBNs Structure matrices

Structure matrices for study entry to entry period and entry period to year 1, similar to Figures 6 and 7, can be found in an accompanying zip file1 for all sites.

## Validation set performance of each DBN model

**Supplemental Table 2:** UCLA+PHS model performance on the validation set for each annual target (i.e., outcome of >=40% eGFR decline). Prediction year: year of prediction using past information prior to the year of prediction.

| Metric | Prediction Year 1, target year 1 | Prediction Year 2, target year 2 | Prediction Year 3, target year 3 | Prediction Year 4, target year 4 | Prediction Year 5, target year 5 | Prediction Year 6, target year 6 |
| --- | --- | --- | --- | --- | --- | --- |
| AUC ROC | 0.67 | 0.72 | 0.79 | 0.81 | 0.83 | 0.85 |
| AP | 0.02 | 0.05 | 0.12 | 0.16 | 0.19 | 0.26 |
| Brier Score loss | 0.02 | 0.03 | 0.03 | 0.03 | 0.02 | 0.01 |
| TNs | 348904 | 383090 | 375700 | 386253 | 386050 | 399490 |
| FPs | 97623 | 62434 | 69247 | 58395 | 58467 | 45403 |
| FNs | 1893 | 2451 | 2178 | 2206 | 1960 | 1755 |
| TPs | 1741 | 2186 | 3036 | 3307 | 3684 | 3513 |
| TNs Rate | 0.78 | 0.86 | 0.84 | 0.87 | 0.87 | 0.90 |
| FPs Rate | 0.22 | 0.14 | 0.16 | 0.13 | 0.13 | 0.10 |
| FNs Rate | 0.52 | 0.53 | 0.42 | 0.40 | 0.35 | 0.33 |
| TPs Rate | 0.48 | 0.47 | 0.58 | 0.60 | 0.65 | 0.67 |
| Precision/PPV | 0.02 | 0.03 | 0.04 | 0.05 | 0.06 | 0.07 |
| Recall/Sensitivity | 0.48 | 0.47 | 0.58 | 0.60 | 0.65 | 0.67 |
| Specificity | 0.78 | 0.86 | 0.84 | 0.87 | 0.87 | 0.90 |
| F1 score | 0.03 | 0.06 | 0.08 | 0.10 | 0.11 | 0.13 |
| Optimal Threshold | 0.1 | 0.18 | 0.17 | 0.19 | 0.15 | 0.03 |
| Outcome Prevalence | 0.81 | 1.03 | 1.16 | 1.22 | 1.25 | 1.17 |

**Supplemental Table 3:** PHS model performance on the validation set for each annual target (i.e., outcome of >=40% eGFR decline). Prediction year: year of prediction using past information prior to the year of prediction.

| Metric | Prediction Year 1, target year 1 | Prediction Year 2, target year 2 | Prediction Year 3, target year 3 | Prediction Year 4, target year 4 | Prediction Year 5, target year 5 | Prediction Year 6, target year 6 |
| --- | --- | --- | --- | --- | --- | --- |
| AUC ROC | 0.67 | 0.72 | 0.79 | 0.80 | 0.82 | 0.83 |
| AP | 0.03 | 0.05 | 0.11 | 0.15 | 0.18 | 0.24 |
| Brier Score loss | 0.02 | 0.03 | 0.03 | 0.03 | 0.02 | 0.01 |
| TNs | 281997 | 322027 | 323781 | 318647 | 340146 | 333273 |
| FPs | 98329 | 57415 | 55100 | 60040 | 38491 | 45616 |
| FNs | 1543 | 2113 | 1926 | 1871 | 1993 | 1677 |
| TPs | 1653 | 1967 | 2715 | 2964 | 2892 | 2956 |
| TNs Rate | 0.74 | 0.85 | 0.85 | 0.84 | 0.90 | 0.88 |
| FPs Rate | 0.26 | 0.15 | 0.15 | 0.16 | 0.10 | 0.12 |
| FNs Rate | 0.48 | 0.52 | 0.41 | 0.39 | 0.41 | 0.36 |
| TPs Rate | 0.52 | 0.48 | 0.59 | 0.61 | 0.59 | 0.64 |
| Precision/PPV | 0.02 | 0.03 | 0.05 | 0.05 | 0.07 | 0.06 |
| Recall/Sensitivity | 0.52 | 0.48 | 0.59 | 0.61 | 0.59 | 0.64 |
| Specificity | 0.74 | 0.85 | 0.85 | 0.84 | 0.90 | 0.88 |
| F1 score | 0.03 | 0.06 | 0.09 | 0.09 | 0.13 | 0.11 |
| Optimal Threshold | 0.1 | 0.17 | 0.18 | 0.18 | 0.16 | 0.03 |
| Outcome Prevalence | 0.83 | 1.06 | 1.21 | 1.26 | 1.27 | 1.21 |

**Supplemental Table 4:** UCLA model performance on the validation set for each annual target (i.e., outcome of >=40% eGFR decline). Prediction year: year of prediction using past information prior to the year of prediction.

| Metric | Prediction Year 1, target year 1 | Prediction Year 2, target year 2 | Prediction Year 3, target year 3 | Prediction Year 4, target year 4 | Prediction Year 5, target year 5 | Prediction Year 6, target year 6 |
| --- | --- | --- | --- | --- | --- | --- |
| AUC ROC | 0.69 | 0.74 | 0.85 | 0.87 | 0.87 | 0.87 |
| AP | 0.02 | 0.05 | 0.15 | 0.22 | 0.26 | 0.28 |
| Brier Score loss | 0.02 | 0.03 | 0.03 | 0.02 | 0.02 | 0.01 |
| TNs | 56898 | 49998 | 51690 | 60531 | 58738 | 56149 |
| FPs | 9240 | 16037 | 14297 | 5489 | 7256 | 9855 |
| FNs | 265 | 213 | 136 | 197 | 161 | 158 |
| TPs | 236 | 391 | 516 | 422 | 484 | 477 |
| TNs Rate | 0.86 | 0.76 | 0.78 | 0.92 | 0.89 | 0.85 |
| FPs Rate | 0.14 | 0.24 | 0.22 | 0.08 | 0.11 | 0.15 |
| FNs Rate | 0.53 | 0.35 | 0.21 | 0.32 | 0.25 | 0.25 |
| TPs Rate | 0.47 | 0.65 | 0.79 | 0.68 | 0.75 | 0.75 |
| Precision/PPV | 0.02 | 0.02 | 0.03 | 0.07 | 0.06 | 0.05 |
| Recall/Sensitivity | 0.47 | 0.65 | 0.79 | 0.68 | 0.75 | 0.75 |
| Specificity | 0.86 | 0.76 | 0.78 | 0.92 | 0.89 | 0.85 |
| F1 score | 0.05 | 0.05 | 0.07 | 0.13 | 0.12 | 0.09 |
| Optimal Threshold | 0.12 | 0.17 | 0.14 | 0.19 | 0.13 | 0.02 |
| Outcome Prevalence | 0.75 | 0.91 | 0.98 | 0.93 | 0.97 | 0.95 |

## UCLA+PHS DBN model comparison against ML models over time

**Supplemental Figure 1:** Comparative AUCROC Performance across a 6-year Prediction Horizon. Area Under the Receiver Operating Characteristic (AUCROC) curve for the proposed dynamic Bayesian network (DBN) compared to standard machine learning models (CatBoost, logistic regression, random forest, and XGBoost). While static models show a performance plateau, the DBN demonstrates a significant upward trend in discriminative power as more longitudinal data and temporal dependencies are integrated, reaching a peak AUCROC of 0.84 by Year 6.


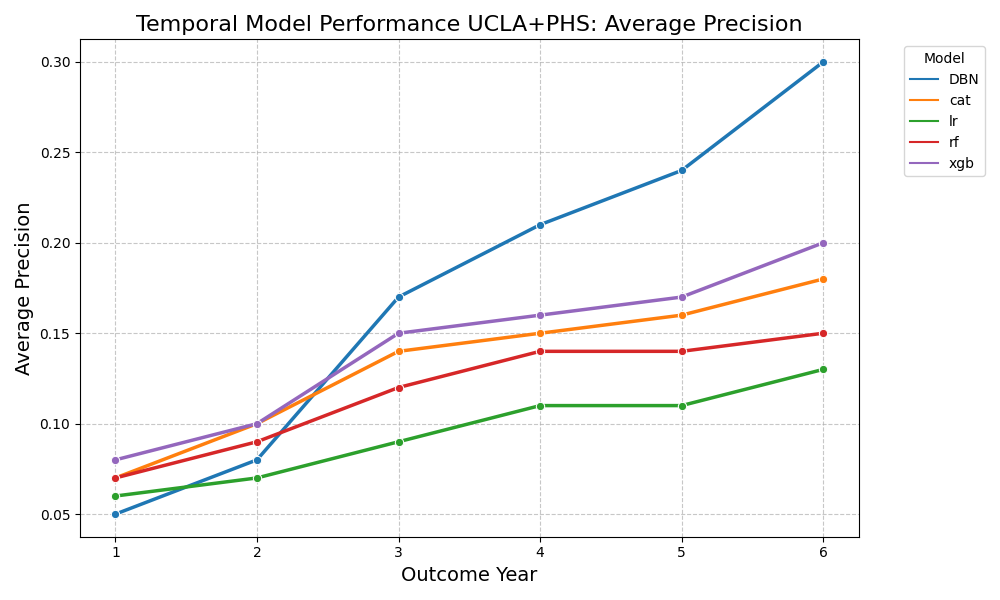


**Supplemental Figure 2:** Comparative Average Precision (AUPRC) across a 6-year Prediction Horizon. Average precision (a surrogate for area under the precision-recall curve) of the DBN against baseline classifiers. The DBN significantly outperforms all static machine learning models as the prediction window extends. By Year 6, the DBN achieves an average precision of 0.30, maintaining superior performance despite the increased data sparsity and eGFR fluctuations inherent in long-term clinical EHR data.


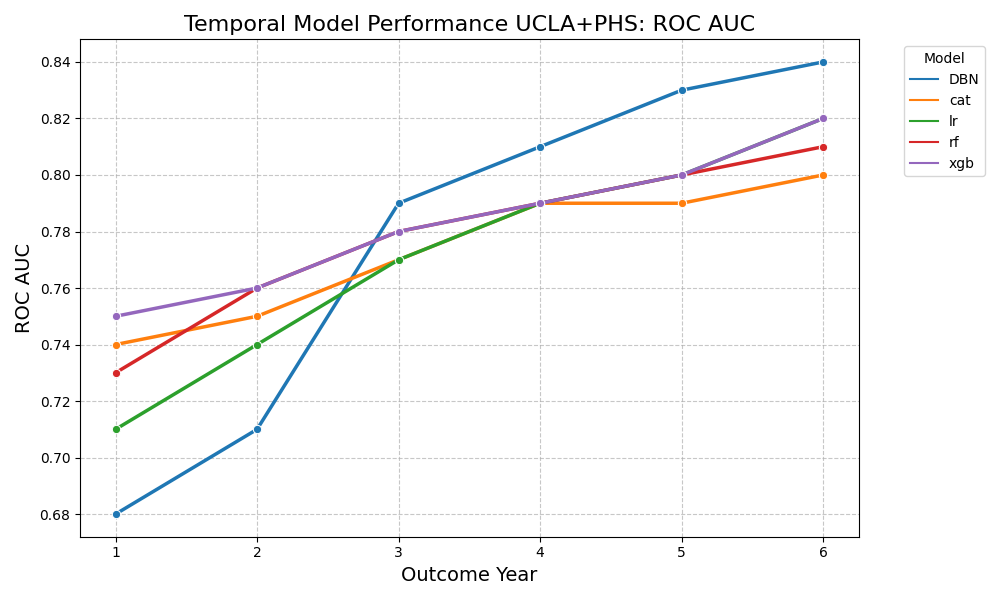


**Supplemental Table 5:** ROC AUC and Average Precision metrics for the DBN against baseline models (CatBoost, Logistic Regression, Random Forest, and XGBoost) over 1, 3, and 6-year horizons. Results are based on external validation using the UCLA and PHS cohorts.

| Target Year | Metric | DBN | cat | lr | rf | xgb |
| --- | --- | --- | --- | --- | --- | --- |
| 1 | roc_auc | 0.68  (0.67-0.68) | 0.74  (0.73-0.75) | 0.71  (0.70-0.72) | 0.73 (0.73-0.74) | 0.75 (0.74-0.76) |
|  | average_precision | 0.05  (0.05-0.05) | 0.07  (0.07-0.08) | 0.06  (0.05-0.06) | 0.07 (0.06-0.07) | 0.08 (0.07-0.08) |
| 2 | roc_auc | 0.71  (0.71-0.72) | 0.75  (0.74-0.76) | 0.74  (0.73-0.75) | 0.76 (0.75-0.77) | 0.76 (0.75-0.76) |
|  | average_precision | 0.08  (0.07-0.08) | 0.10  (0.09-0.11) | 0.07  (0.07-0.08) | 0.09 (0.09-0.10) | 0.10 (0.10-0.11) |
| 3 | roc_auc | 0.79  (0.78-0.79) | 0.77  (0.77-0.78) | 0.77  (0.77-0.78) | 0.78 (0.78-0.79) | 0.78 (0.77-0.78) |
|  | average_precision | 0.17  (0.16-0.17) | 0.14  (0.13-0.15) | 0.09  (0.09-0.10) | 0.12 (0.12-0.13) | 0.15 (0.14-0.15) |
| 4 | roc_auc | 0.81  (0.80-0.81) | 0.79  (0.78-0.79) | 0.79  (0.78-0.79) | 0.79 (0.78-0.80) | 0.79 (0.79-0.80) |
|  | average_precision | 0.21  (0.20-0.22) | 0.15  (0.14-0.16) | 0.11  (0.10-0.11) | 0.14 (0.13-0.15) | 0.16 (0.15-0.17) |
| 5 | roc_auc | 0.83  (0.83-0.84) | 0.79  (0.78-0.80) | 0.80  (0.80-0.81) | 0.80 (0.80-0.81) | 0.80 (0.80-0.81) |
|  | average_precision | 0.24  (0.24-0.25) | 0.16  (0.15-0.17) | 0.11  (0.11-0.12) | 0.14 (0.13-0.15) | 0.17 (0.16-0.18) |
| 6 | roc_auc | 0.84  (0.84-0.85) | 0.80  (0.80-0.81) | 0.82  (0.82-0.83) | 0.81 (0.80-0.81) | 0.82 (0.81-0.82) |
|  | average_precision | 0.30  (0.29-0.31) | 0.18  (0.17-0.19) | 0.13  (0.13-0.14) | 0.15 (0.14-0.16) | 0.20 (0.19-0.21) |

## EM algorithm parameters

**Supplemental Table 6:** DBNs’ training parameters.

| Parameter Category | Specification | Optimal Value / Method |
| --- | --- | --- |
| Structure Learning | Primary Algorithm | Ranking Approaches for Unknown Structures (RAUS, Gordon et al.,) [21] |
| Expectation-Maximization Algorithm | Implementation | PySMILE Learning EM |
|  | Initialization | Parameters uniformized before learning (set_uniformize_parameters(True)) |
|  | Seed/Randomization | Fixed seed (set_seed(0)); no randomization (set_randomize_parameters(False)) |
|  | Hyperparameters | Equivalent Sample Size set to 0; Relevance-based learning enabled |

## Dynamic Nature of Kidney Function in EHR Data

A decline in eGFR of ≥40%, in the UCLA and PHS combined dataset, is frequently not a terminal event but rather a fluctuating surrogate marker. Evidence of non-terminality and recovery is provided as follows: The data below (Tables 7-9) demonstrate significant upswings in kidney function, even after the ≥40% decline threshold is met.

**Supplemental Table 7:** Annual Significant eGFR Upswings (>20%)

| Time Interval | Count of Significant Upswings (>20%) |
| --- | --- |
| Year 0 to Year 1 | 111,694 |
| Year 1 to Year 2 | 49,304 |
| Year 2 to Year 3 | 43,009 |
| Year 3 to Year 4 | 38,890 |
| Year 4 to Year 5 | 32,450 |
| Year 5 to Year 6 | 27,060 |
| Total (6 Years) | 302,407 |

**Supplemental Table 8:** Recovery Rates Among Recurrent Decliners (Functional Upswings)

| Recurrence Level | Total Patients with Recurrence | Patients with >20% Upswing in eGFR (Recovery) | Recovery Rate |
| --- | --- | --- | --- |
| >1 Decline Event | 50,144 | 11,962 | 23.86% |
| >2 Decline Events | 25,473 | 6,453 | 25.33% |
| >3 Decline Events | 14,228 | 3,699 | 26.00% |
| >4 Decline Events | 8,162 | 2,141 | 26.23% |

**Supplemental Table 9:** Recovery to Baseline (>60% of Baseline eGFR)

| Recurrence Level | Total Patients with ≥40% Decline | Patients Recovered to >60% of Baseline eGFR | Recovery Rate |
| --- | --- | --- | --- |
| ≥1 Decline Event | 146,043 | 13,787 | 9.44% |

**Supplemental Table 10:** Recovery Rates Specifically Among Recurrent Decliners

| Recurrence Level | Total Patients with Recurrence | Recurrent Patients Recovered to >60% Baseline | Recovery Rate |
| --- | --- | --- | --- |
| >1 Decline Event | 50,144 | 8,277 | 16.51% |
| >2 Decline Events | 25,473 | 3,942 | 15.48% |
| >3 Decline Events | 14,228 | 2,080 | 14.62% |
| >4 Decline Events | 8,162 | 1,121 | 13.73% |

1. https://zenodo.org/records/15851525 [↑](#footnote-ref-1)
